# Insane Asylums

**Published:** 1850-02

**Authors:** 


### INSANE ASYLUMS.

Efforts are making in Michigan for the erection of an Asylum for the Insane, at Kalamazoo. The Detroit Tribune, in speaking of this movement, justly remarks that, "in respect to Insane Asylums, Philadelphia was the pioneer."

The Pennsylvania Hospital, having an insane department, was founded in 1752, and is the oldest in the country. For eighty-nine years patients were received at the Institution in the city, during which time 4367 were admitted. In the year 1841, the new building in Blockley township having been completed, the insane patients were removed thither, from which time, until the commencement of 1849, there were received (including those transferred to it from the city) 1391 patients. The whole number received in the period, from the foundation of the Hospital till January, 1849, is 5647. The total number in the "Pennsylvania Hospital for the Insane," during the year 1848, was 403. The highest number was 208, the lowest 188; and the average number under treatment during the entire period was 199. This Institution is now capable of accommodating 220 patients with their attendants, and of a classification of them into eight distinct divisions of each sex, with the fixtures and conveniences required for their proper custody and treatment.

There is another Institution at Frankford, near Philadelphia, under the management of the Society of Friends, capable of accommodating about 80 patients. The Philadelphia Almshouse has also recently undergone extensive modifications and improvements, under the supervision of the principal physician, Dr. Benedict, (recently appointed successor to Dr. Brigham,) which have placed it upon a footing with the best institutions in the country.

These Institutions, together with the State Asylum at Harrisburg, now building, will in the course of a very short time enable Pennsylvania to accommodate upwards of 1000 insane patients, a fact which cannot be otherwise than gratifying to every benevolent mind, when it is remembered that for those who are visited with insanity, be they rich or poor, a hospital is the only resource.

